# Editorial: The role of *Helicobacter pylori* in gastric carcinogenesis

**DOI:** 10.3389/fonc.2023.1233890

**Published:** 2023-06-26

**Authors:** Javier Torres, Rui M. Ferreira, Ikuko Kato

**Affiliations:** ^1^Unidad de Investigacion en Enfermedades Infecciosas, Unidad Medica de Alta Especialidad (UMAE) Pediatria, Instituto Mexicano del Seguro Social, Ciudad de Mexico, Mexico; ^2^Instituto de Investigação e Inovação, Universidade do Porto (i3S), Porto, Portugal; ^3^Institute of Molecular Pathology and Immunology of the University of Porto (Ipatimup), Porto, Portugal; ^4^Department of Oncology, Wayne State University, Porto, Portugal; ^5^Department of Pathology, Wayne State University, Detroit, MI, United States

**Keywords:** *Helicobacter pylori*, gastric cancer, oncogenesis, evolution, gastric mucosa

*H. pylori* (*Hp*) is the only bacterium recognized as a cancer-causing agent by the WHO and has drawn attention from several disciplines, including oncology, microbiology, immunology, cell biology, epidemiology, genomics, and even human evolution. Evidence suggests that *Hp* has colonized the human stomach since the origin of our species, and it is not surprising that this bacterium mirrors the race of its human host (1). This marked human-bacterium co-evolution implies a symbiotic relationship in which both benefit from sharing a highly specialized niche. *Hp* represents the best- documented member of the human microbiota that has co-evolved with humans. While more than 60% of the adult population remains colonized, less than 2% of those colonized will eventually develop gastric cancer (GC), strongly suggesting that GC is a rare event occurring only under as yet unknown conditions.

Colonization of the gastric mucosa by *Hp* elicits an inflammatory reaction, a natural dialogue between the two species, as seen with the gut microbiota. The chronic inflammation induced by *Hp* significantly alters the transcriptomic pattern of the gastric mucosal cells, which in turn alter the *Hp* transcriptome. When this relationship lasts for decades , as with human-*Hp*, accidents can happen, giving rise to a hostile relationship that threatens homeostasis. The challenge for science is to understand the natural history of this relationship.

In this Research Topic, five groups of researchers present results that add to our knowledge of the human-*Hp* dialog, particularly when things go wrong, and aim to understand the mechanisms behind tissue damage. He et al. exposed gastric cell lines to *Hp* lysates for 30 generations and showed that this prolonged exposure to *Hp* antigens leads to the promotion of cell proliferation, and inhibition of apoptosis and autophagy, probably *via* the Nod1-NF-kB/MAPK-ERK/FOXO4 pathway. Infection of gerbils with *Hp* for 90 weeks produced similar results. Thus, chronic exposure to *Hp* seems to modify the response of gastric epithelial cells in a way that favors the development of gastric preneoplastic lesions.

Jan et al. studied patients with GC and *Hp* infection and found that infection and *cagA* expression is associated with increased expression of IL-6, IL-10, and TGF-β, and with activation of the JAK/STAT pathway *via* inactivation of the promoter of SOCS1 by hypermethylation of its promoter region. The authors found that *Hp* is associated with unregulated inflammation even when patients have advanced GC, an observation that challenges the current notion that when GC develops, *Hp* is absent or has extremely low metabolic activity. If *Hp* keeps pushing boundaries at this late clinical-stage, then *Hp* eradication should be considered even in GC cases.

The histone demethylase JARID1B was investigated in GC tissues by Zheng et al., who found that increased expression has a significant correlation with poor survival, low immune score, low stromal score, and increased proliferation of GC cells, probably by regulating the expression of CCND1. Furthermore, *in vitro* and *in vivo* experimental *in vitro in vivo* models showed that *Hp* upregulates the expression of JARID1B by downregulation of miR-29c, a regulator of JARID1B. Histone demethylases have been shown to have a role in tumorigenesis in breast cancer, ovarian cancer, colorectal , and even lung cancers (2). This study offers another explanation for the role of *Hp* in causing dysregulated gene expression.

Angiogenesis is an essential component in all cancers to provide nutrients and oxygen to tissue with highly unregulated growth. Previous studies have documented the ability of *Hp* to activate factors that stimulate angiogenesis. Malespin-Bendaña et al. extended these studies by documenting the role of *Angpt2*, *Vegf-A*, and *Tnf-A* in angiogenesis using a mouse model. It is known that *Hp* activates NF-κβ, which induces the expression of TNF-A, which then upregulates the expression of *Vegf-A*, *Angpt2*, and *Tie2*. The mouse model demonstrates that *Hp* can also downregulate *Angpt1*. Thus, *Hp* can activate and sustain angiogenesis to feed unwanted cells.

The anatomic geography of *Hp* colonization has been under studied and may provide important clues to understanding how *Hp* lives in intimate contact with different cell types in the gastric mucosa. Beccaceci and Sigal reviewed the few studies that have addressed this issue and offered a challenging hypothesis about the consequences of *Hp* colonization along the gastric glands. Contrary to previous theories, *Hp* has evolved to live deep in the glands in close contact with stem cells, a condition that may help *Hp* thrive in the stomach for decades but also carries the risk of damaging the progenitor cell sanctuary.

*Hp* can modify the dialog between different regions of the gland from the tip to the base regions, including differentiated epithelial , progenitor, stem, and stromal cells, with consequences for cell cycle, differentiation, proliferation, and other functions necessary for homeostasis and the inflammatory-immune response. Most cells in the glands have a short life cycle, and it is difficult to assume that they play a major role in oncogenesis. In contrast, effects on stem cells can have long-lasting effects and are likely to be more important in oncogenesis.

In the 40 years since the discovery of *Hp*, much has been learned about its physiology, which is highly specialized for living in the human gastric mucosa, a niche with extremely harsh conditions for most other microorganisms. This exquisite adaptation is the result of human-*Hp* co-evolution since the beginning of our species. This VIP resident of the gastric mucosa has learned to offer health benefits to its host, as expected for key components of the human microbiota (3). *Hp* has also been recognized as the main risk factor for GC, a fact that might be related to changes in our ways of living. Early humans lived as nomads, rather isolated, whereas now we live in large cities. This social evolution (sanitation, diet, personal habits, medicine, etc.) has been faster than biological evolution, resulting in a much longer life expectancy and a longer human-*Hp* relationship, with unexpected circumstances that increase the chances of biological accidents leading to disease (**Figure 1**). Understanding the conditions under which these accidents happen will provide tools to prevent unwanted outcomes and preserve the benefits gained from human-*Hp* symbiosis.

**Figure 1:**
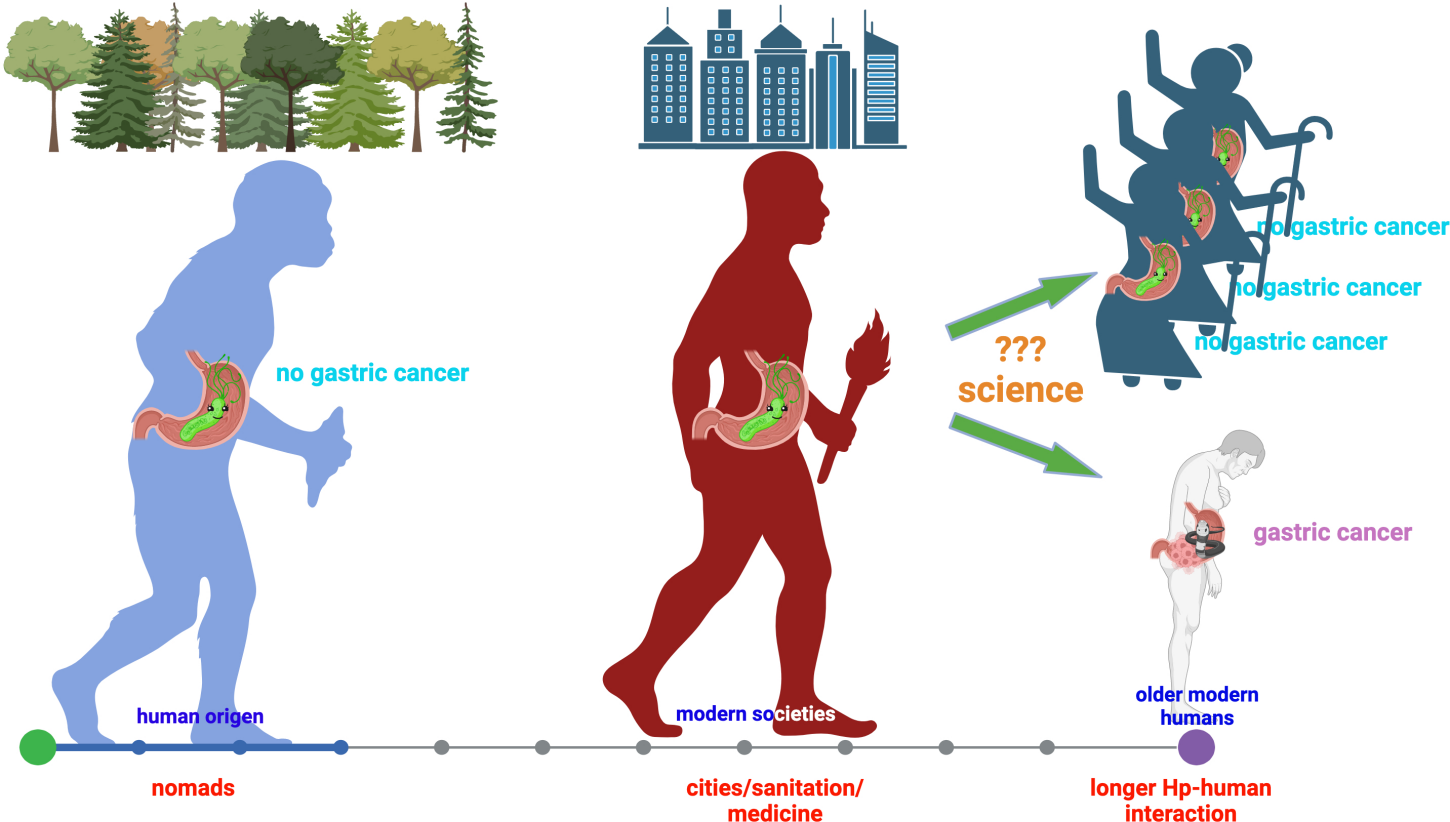
Human-*H. pylori* co-evolution, social evolution, and gastric cancer risk. This figure was designed using BioRender.

## Author contributions

JT wrote the initial draft and reviewed the final manuscript. RF reviewed and edited the manuscript. IK reviewed and edited the manuscript. All authors contributed to the article and approved the submitted version.
